# The Evolvability of Words: On the Nature of Lexical Items in Minimalism

**DOI:** 10.3389/fpsyg.2019.03071

**Published:** 2020-01-24

**Authors:** Brady Clark

**Affiliations:** Department of Linguistics, Northwestern University, Evanston, IL, United States

**Keywords:** lexical semantics, language evolution, words, lexical items, anti-individualism, individualism, internalism/externalism

## Abstract

Work within the minimalist program attempts to meet the criterion of evolvability: “any mechanisms and primitives ascribed to UG rather than derived from independent factors must plausibly have emerged in what appears to have been a unique and relatively sudden event on the evolutionary timescale” (Chomsky et al., 2017). On minimalist assumptions the evolution of the language faculty must have involved at least three major developments: (i) the evolution of computational atoms, lexical items, understood as bundles of features, (ii) the evolution of a single, simple recursive operation that glues together lexical items and complexes of lexical items, and (iii) externalization linking the syntactic component of the language faculty to the cognitive systems that humans use for sound and gesture. The first development, the evolution of lexical items and the lexicon, is especially poorly understood. A complete account of the evolution of lexical items will state what evolved, how, and why. The focus of this article is the first question: what evolved. What properties do lexical items have, what determines these properties, and what is the internal structure of lexical entries? The article identifies what the key open problems are for a minimalist account of the evolution of words that strives to meet the criterion of evolvability.

## 1. Introduction

The minimalist program (henceforth, *minimalism*; see Chomsky, 1995b, 2016; Marantz, 1995; Belletti and Rizzi, 2002; Boeckx, 2006; Hornstein, 2009; Berwick and Chomsky, 2016)^1^ developed out of the Principles and Parameters approach to syntax. Minimalism explores the idea that the basic operations of the human language faculty are simple and few, and that the attested complexities of natural language (such as unbounded dependencies) are a byproduct of the interactions of subsystems. This view of the language faculty attempts to meet what some have called the *criterion of evolvability*: “any mechanisms and primitives ascribed to UG rather than derived from independent factors must plausibly have emerged in what appears to have been a unique and relatively sudden event on the evolutionary timescale” (Chomsky et al., 2017).

On minimalist assumptions the evolution of the language faculty must have involved at least three major developments (see, e.g., Berwick, 2011; Berwick and Chomsky, 2016)^2^:

- evolution of lexical items understood as bundles of features
- evolution of a single, simple recursive operation, Merge, that glues together lexical items and complexes of lexical items, thus forming larger units
- externalization linking the syntactic component of the language faculty to the cognitive systems that humans use for sound and gesture.

Each of these developments must be part of any minimalist account of language evolution. One cannot be reduced to the other. Berwick (1998) states this explicitly: “Merge cannot tell us everything we need to know. **It does not say how words came to be**, and will have little to say about the word features particular to each language” [emphasis added–BC]. But these three developments are interdependent. For example, as Piattelli-Palmarini (2010, p. 160) argues, if lexical items are defined as mergeable form-meaning pairs, then there could not be any lexical items without syntax because “[w]ords are fully syntactic entities.”

The evolution of lexical items (and the lexicon) is poorly understood. As Chomsky et al. (2017) put it, “[t]he evolutionary origins of … the lexicon and its atoms with all their semantic intricacy … remain a deep mystery.” A complete account of the evolution of lexical items will state what evolved, how, and why. Here I focus primarily on the first issue: what evolved. What properties do lexical items have, what determines these properties (factors entirely internal to the individual?, extramental factors?, some of both?), and what is the internal structure of lexical entries? After a brief discussion of, in very broad strokes, how lexical items are treated in (some varieties of) minimalism, I focus my attention on just one aspect of lexical items: their semantic properties.

Any account of the evolution of words within minimalism needs to address a large number of issues, even if we restrict our attention to just the semantic properties of lexical items. Some of these issues are fairly abstract ones involving the nature of lexical semantic features and their interrelationship; others are more specific issues that pertain to the particular psychological mechanisms that ground the relation between semantic features and the extramental world. I try to get clear on what the core issues are and what tools and evidence we need to address them. The implications of this investigation for a minimalist account of language evolution that strives to meet the criterion of evolvability are severe. Much needs to be clarified about the nature of lexical items and their relationship to other cognitive structures before we can make progress on understanding how words evolved and why. (Noam Chomsky (p.c.) observes correctly that many of the issues that I raise below are not particular to minimalism. While the context for the discussion that follows is minimalist conceptions of lexical items and the lexicon, the issues that I discuss are issues for many other frameworks as well.)

A few caveats. (i) In what follows, I do not present or advocate for (as far as I can tell) a novel account of lexical items within minimalism. (ii) For the most part, I describe some characterizations of lexical items that have appeared in the minimalist literature to get a handle on what evolved in the evolution of words. I review ideas from various accounts, but draw primarily from the expositions of the lexicon and lexical items that have appeared in Chomsky's work (e.g., Chomsky, 1995b, 2000, 2003b, 2016). I make no attempt to be exhaustive, even of Chomsky's work on the topic, nor do I go into much detail about the empirical consequences of various views of the lexicon. These consequences are explored in detail in the work cited throughout this article. (iii) I do not discuss theories of the lexicon, features, or feature structures that have appeared in frameworks embedded within traditions other than the Principles and Parameters approach (e.g., Head-driven Phrase Structure Grammar; see, e.g., Pollard and Sag, 1994).

## 2. Lexical Items and Their Features

As noted in the previous section, minimalist discussions of language evolution have proposed that there were three key developments in the evolution of the language faculty. One development was the emergence of a capacity to construct an infinite range of hierarchically structured expressions through an operation, Merge. This capacity is what (Chomsky, 2016, p. 4) calls the *Basic Property*. Another was the development of the atoms of computation that, when combined, yield those hierarchically structured expressions. I call these computational atoms, when associated with phonological properties, *lexical items* (henceforth, LIs).

The third development is externalization. Chomsky (2016, p. 41), among others, says we should distinguish the emergence of “word-like objects” without phonological properties from the evolutionary development of externalization. Externalization is the mapping of the expressions generated by the syntactic component of the language faculty to the cognitive systems that humans use for sound and gesture (the sensorimotor interface): “When the beneficial mutation [giving rise to Merge—BC] has spread through the group, there would be an advantage to externalization, so the capacity would be linked as a secondary process to the sensorimotor system for externalization and interaction, including communication as a special case” (Berwick and Chomsky, 2011, p. 36).

Characterizations of externalization typically assume that the sensorimotor systems linked to the language faculty are evolutionarily ancient, systems that were largely (perhaps entirely) in place before the development of Merge and the development of LIs. The sensorimotor systems, it is claimed, have little to do with language and have not evolved in any significant way subsequent to the emergence of the faculty of language (Chomsky, 2017, p. 298; Huybregts, 2017). On this view, the articulation of language, through sound, gesture, etc., is considered to be an ancillary aspect of language. Huybregts (2017) presents conceptual and empirical arguments in favor of the view that externalization occurred subsequent to the development of Merge.

Huybregts (2017, p. 292) claims that “externalization may not have required much, or any, further evolution of language.” Externalization is typically characterized as a single evolutionary development. This is likely an oversimplification of this aspect of language evolution. Externalization involved at least the development of mental representations of phonological structure and the linkage of LIs (presumably comprising, prior to externalization, solely semantic and syntactic features) with these representations. There is much more to explore here, but, given the goals of this article, I set aside the issue of externalization. [Tallerman (2014) presents a thorough critique of the standard take on externalization; Jackendoff (2011, p. 616) argues against the notion of externalization presented in Chomsky (2010)].

I assume throughout what follows that the signals that characterize non-human animal communication systems (Hauser, 1996 for an overview) are different from LIs in many respects (see, e.g., Deacon, 1997; Hurford, 2007). (This is not to say that there is no relationship between the properties of non-human animal communicative behavior and the meanings associated with LIs. Bar-On (2018) argues that communicative expressive behaviors displayed by both humans and non-human animals play an important explanatory role in understanding the origins of linguistic meaning.) Consider alarm calls (e.g., vervet monkey alarm calls; see Cheney and Seyfarth, 1990). They are typically indexical (bound to the currently occurring situation): “Alarm calls are about the here and now—or the almost here and now” (Skyrms, 2010, p. 28, 29). The production of alarm calls involves little or no calculation of the attentive state of other animals. They are largely innate. Alarm calls are *functionally referential* (Hauser, 1996, p. 509)^3^. As Deacon (1997, p. 57) puts it: “Alarm calls refer to objects the way laughter does, not the way words do.”

Further, there are a range of concepts that are expressed by LIs (and complexes of LIs) but, as far as we know, are not externalized by non-human animal signals:

Other animals do not create external public representation of quantifiers, sortals, epistemic states, causality, and so on. Other animals may represent their world in terms of such concepts, but they do not communicate about such things (Carey, 2009, p. 464).

As Tallerman (2014, p. 208) observes, some of the externalized concepts that characterize human language may “have developed *through* the use of (externalized) language,” subsequent to the externalization step in language evolution described above. As Hurford (2012, p. 153; cited in Tallerman, 2014) puts it: “public use affects private concepts.”

Contrasts between LIs and non-human animal signals could probably be multiplied indefinitely. For example, some linguistic representations are merely objectual, others are purely objective (Taylor, 2019, p. 113–116). Objective representations stand for real properties and objects. Merely objectual ones (e.g., *Sherlock Holmes*) are “fit” for the job of standing for real existents or real properties but don't. There does not seem to be a robust counterpart to the objectual/objective distinction in non-human animal communication systems.

There is often a great deal of controversy surrounding any claim about non-human animal communication systems, especially with respect to meaning (see, e.g., Scott-Phillips, 2015; Moore, 2016, for a recent exchange regarding meaning in great ape communication). But I take the claim that LIs are vastly different from non-human animal signals to be an uncontroversial one.

Minimalism assumes that each LI comprises properties involved in form (sound, gesture, etc.) and meaning (Chomsky, 1995b, 2000, 2003b; Collins and Stabler, 2016 present a formalization of minimalist syntax). These properties are often referred to as *features*. Universal Grammar provides three sets of features: phonological features (such as VOICE), semantic features (such as CAUSE), and syntactic features (such as category information). Syntactic features are involved in the computational processes (e.g., applications of Merge) that yield hierarchical complexes of LIs. Each LI is a triple 〈SEM, SYN, PHON〉 (Collins and Stabler, 2016, p. 44). SEM and SYN are (possibly empty) subsets of the set of semantic features and the set of syntactic features provided by Universal Grammar. PHON is a string of segments, possibly null, where each segment is a bundle of features (like VOICE). A lexicon is a finite set of LIs^4^.

The lexicon has a number of properties that distinguish it from the sets of signals (such as a set of alarm calls) that characterize non-human animal communication systems. Tallerman (2014, p. 209, 210) discusses some of these properties and their implications for accounts of language evolution. For example, unlike many non-human primate call systems, the lexicon is acquired entirely in ontogeny. Further, the lexicon is very large in size relative to the size of call systems. The capacity to acquire a lexicon presumably involved cognitive changes in the hominin lineage. These changes require an evolutionary account. In what follows, my focus will be LIs (their properties, how those properties are determined, etc.) rather than the lexicon *per se*. But any adequate model of the evolution of words in minimalism will need to incorporate an account of the evolutionary development of the lexicon.

Any minimalist characterization of LIs must answer the following questions:

- What sorts of properties are associated with LIs?
- How are these properties determined? What makes it the case that an LI in the lexicon of a particular individual is an LI with its particular properties?^5^
- What is the internal structure of lexical entries?

In what follows, I address each of these questions, focusing on lexical semantic properties and the internal structure of the semantic component of LIs. The goal will be to clearly articulate what minimalist accounts of the evolution of words must explain.

### 2.1. The Inventory of Semantic Features

As discussed in the introduction, most work in minimalism assumes that LIs have features and that some of these features are semantic ones. In what follows, I make a few (tendentious) assumptions about semantic features. As noted in the introduction to this section, a set of lexical semantic features is just one of several components of each LI, alongside a set of syntactic features and a set of phonological features. I assume that many semantic features (like CAUSE, CONTACT, MANNER OF MOTION, ANIMATE, etc.) correspond to concepts in cognitive faculties outside language cognition. (As noted below, some of these concepts, like BODY, may be shared by humans and non-human animals.) These semantic features are concepts that have been coopted for lexical representation, a claim that I return to below. (I use capitals throughout for the names of both semantic features and concepts.)

Following Glanzberg (2011), among others, I do not assume that LIs simply express concepts, though they are associated with them (in some fashion). The relationship between LIs, semantic features, lexical meanings, and concepts is a complex one. I do assume that concepts are mental representations that are not necessarily specific to language cognition. Following Fodor (1975, 1998; 2008, among many other publications), I take the concepts that humans externalize with language to be “composable mental symbols with which thinkers can think about things” (Pietroski, 2018, p. 348). There is overwhelming evidence that both humans and many non-human animal species have concepts (Hurford, 2007; Carey, 2009), but the conceptual repertoire varies from species to species and composability appears to be unique to human cognition.

There is widespread disagreement about the ultimate basis for concepts and their internal structure^6^. I don't take a strong position on either of these issues here, although I touch on them below. Tallerman (2014, p. 208) observes that in minimalism, “what is meant by ‘conceptual atoms’ is some set of basic concepts which either constitute, form a part of, or are precursors to lexical items”^7^. Tallerman points out that these are three distinct possibilities. Chomsky (2016, p. 41) distinguishes the atoms of computation (or, as he puts it, “atomic concepts”) from words and lexical items, although these terms (“atoms,” “words,” “lexical items”) are sometimes used interchangeably. Computational atoms are the elements that language uses, through Merge, to construct an infinite range of hierarchically structured expressions. These atoms connect to the conceptual-intentional interface for mental processes (Chomsky, 2016, p. 4) but they do not necessarily have phonological properties. LIs, in contrast, are computational atoms that have been assigned phonological properties through externalization, sound being just one possible modality. (Because I have set aside the issue of externalization, I do not attempt to systematically distinguish between LIs and computational atoms in what follows.)

Features alone determine the identity of LIs: “any feature change yields a different LI” (Chomsky, 2003b, p. 265). As I discuss below, according to minimalism, word meaning is determined by some combination of semantic features provided by Universal Grammar (many of which were ultimately coopted from other areas of cognition) and meaning-related properties drawn from cognitive structures outside of language cognition: “word meaning and the knowledge associated with it may include several sorts of structures”—conceptual, visual, auditory, etc.—each structure playing a role in thought (Jackendoff, 2012, p. 125). That word meaning is determined by some combination of linguistic and non-linguistic properties is an assumption shared widely by linguists (e.g., Chomsky, 1975; Jackendoff, 2012), philosophers (e.g., Glanzberg, 2014, 2018; Taylor, 2019), and psychologists (e.g., Rips, 2011).

Ultimately, according to minimalism, the meaning-related properties associated with linguistic expressions generated by the language faculty are “information that is used by conceptual-intentional systems to engage the world in different ways as the language user thinks and talks in terms of the perspectives made available by the resources of the mind” (Chomsky, 2003b, p. 273). These properties provide a certain constrained range of perspectives for referring to aspects of the world (Chomsky, 2000, p. 36, 2016, p. 50; see also Borg, 2012, p. 149; Stainton, 2006, p. 924 for related discussion). They “focus attention on selected aspects of the world as it is taken to be by other cognitive systems, and provide intricate and highly specialized perspectives from which to view them, crucially involving human interests and concerns even in the simplest cases” (Chomsky, 1995a, p. 20). For example, consider the complexities associated with the word *near*: if I text you *I'm near your house*, you will be surprised if you then turn around in your living room and find me standing right there (as opposed to learning that I'm just up the block).

What meaning-related properties are typically assumed to be encoded by LIs? Most accounts of LIs assume that semantic features tell us something about “the worldly object, property, or event that is the assigned semantic value of the relevant expression” (Taylor, 2019, p. 29). But, as mentioned above, semantic features do not represent our total knowledge of the object, property, or event that correspond to the semantic value of individual LIs:“[l]inguistic theory is not the whole theory of human knowledge” (Higginbotham, 1989a, p. 470).

The semantic features of LIs that have been mentioned in the literature are eclectic. I will not attempt to survey them here. As I noted earlier, it would be premature to present a putatively complete inventory of lexical semantic features, given the lack of consensus within minimalism about the right theory of features or their key properties. I will instead point to some of the proposals in the literature to give a sense of the range of semantic features that have been introduced. Then I try to identify some generalizations about these features.

Collins and Stabler (2016, p. 44) list semantic features “pertaining to aktionsart, thematic roles, negation, focus, topic, tense, aspect, quantification, definiteness, plurality, causation.” These features are a motley crew, relating to the internal structure of eventualities, the semantic roles of eventuality participants, discourse properties, and numerical notions. Throughout his work, Chomsky has emphasized the rich range of properties, both concrete and abstract, that can be involved in fixing word meaning—lexical features indicating semantic role (such as AGENT, INSTRUMENT, and GOAL)^8^, semantic relations between words, and properties of quantifying determiners and anaphora. These meaning-related properties are “expressed in part on the level of semantic representation separate from extralinguistic considerations” (Chomsky, 1979, p. 141, 142)^9^. On this view, particular LIs will be associated with meaning-related properties that are some combination of intralinguistic properties and information about the semantic value of the expression. For example, the lexical entry for the common noun *book* indicates that it is a nominal (rather than verbal) expression used to refer to an artifact, rather than an expression used to refer to a substance like water or a pure abstraction like loyalty, with both material and abstract characteristics (Chomsky, 2000, p. 15, 16).

Semantic features encoded in lexical items are (like phonological features and syntactic features) assumed in minimalism to be part of our biological endowment. They are provided by Universal Grammar, but may be unrealized: individuals and languages differ in what semantic features are involved in fixing meaning (Chomsky, 2003b, p. 277).

The semantic features encoded in lexical entries are assumed to be innate but it is possible that not all lexical semantic properties are provided by the language faculty alone. Some may be drawn from other faculties of the mind such as a “non-linguistic system of common sense understanding” (Chomsky, 1975, p. 42), “a system of beliefs and expectations about the nature and behavior of objects” (Chomsky, 1975, p. 139). That is, lexical meaning is an interface phenomenon, pulling from multiple areas of cognition. Individual LIs are intersectional, “located in a ‘semantic space’ generated by the interaction of the language faculty and other faculties of the mind” (Chomsky, 1975, p. 42). For example, the semantic value of the common noun *tiger* is a “function of the place of the associated concept in the non-linguistic system of common-sense understanding…though the linguistic system may provide some more abstract semantic properties” (Chomsky, 1975, p. 42).

I take the claim in the previous paragraph (that the semantic properties associated with LIs are intersectional, drawing from a range of cognitive systems) to be an uncontroversial one, but one that makes the task of determining which semantic properties are encoded in LIs, and which are not, challenging. It has long been observed that it is very difficult to determine the line, if any, that separates knowledge of linguistic meaning (expressed as semantic features of LIs in minimalism), strictly speaking, from all-inclusive knowledge of the world, both mental and extramental (Chomsky, 1979, p. 142, 2000, p. 15; Fodor, 1998, p. 44–46; Higginbotham, 1989a, p. 470–471; Taylor, 2019, p. 31). Chomsky (2000, p. 15), discussing the word *book*, observes that there is no good way currently to determine whether a semantic property is part of the lexical meaning of the word (i.e., a semantic feature) or instead attached to the concept associated with the word^10^. In fact, we may be unable, in practice and in principle, to distinguish encyclopedic, worldly knowledge from strictly lexical semantic knowledge, if Quine is correct about our inability to separate convention from fact:

The lore of our fathers is a fabric of sentences. In our hands it develops and changes, through more or less arbitrary and deliberate revisions and additions of our own, more or less directly occasioned by the continuing stimulation of our sense organs. It is a pale gray lore, black with fact and white with convention. But I have found no substantial reasons for our concluding that there are any quite black threads in it, or any white ones (Quine, 1956, p. 86, 87, quoted in Taylor, 2019, p. 31).

Language change, specifically semantic change (change in the meaning of words over time such as amelioration and pejoration), might give us a handle on the distinction between the lexical semantic features of LIs and encyclopedic, meaning-related properties associated with other cognitive faculties. While careful to point out that there isn't a sharp boundary between the different classes of properties associated with LIs, Taylor (2019, p. 167, 168) distinguishes lexical change (e.g., the development from a deontic/obligation interpretation, as in *you must do X*, of an epistemic interpretation, as in *X must be the case*, common to many modal verbs—like the English auxiliary *must*—cross-linguistically) from encyclopedic change, proposing that semantic properties provided by faculties of the mind other than the language faculty might include an evolving set of metaphysical details about the object, property, or event expressed by a particular LI such as *book*. In contrast, semantic features encoded by the lexicon are “to some degree insulated from pressure to change merely as a consequence of our ever-increasing knowledge of the world” (Taylor, 2019, p. 167). If this is on the right track, the way in which different lexical semantic properties behave during language change might help us pinpoint the semantic features encoded in LIs.

Another factor that might help us distinguish encyclopedic knowledge from strictly lexical semantic knowledge when examining a meaning-related property of a linguistic expression is the interaction between that property and other areas of grammar, particularly morphosyntax. An assumption of most work on lexical semantics (see Glanzberg, 2018, p. 205 for references) is that what is crucial to language design and linguistic theory is not so much the distinction between knowledge of (linguistic) meaning and non-linguistic encyclopedic knowledge, but rather a distinction between semantic properties that have systematic morphosyntactic effects and semantic properties that do not (Higginbotham, 1989a, p. 470; Borg, 2012, p. 199)^11^. For example, a condition on middle formation (a construction in which the patient argument of a verb is realized as the subject and the agent is unexpressed) appears to be that the affected argument is construed as physically altered by the action expressed by the verb (Higginbotham, 1989a, p. 471). Compare (1) and (2):

(1) That bread cuts easily
(2) #That bread taps easily

Why certain concepts but not others are co-opted as morphosyntactically potent semantic features (like AGENT or CAUSE) of LIs is unclear, but the recruitment of these concepts as semantic features within the lexicon is taken to explain how and why they impact the morphosyntactic distribution of the LIs they are associated with. As noted above, most accounts of lexical semantics assume that meaning-related information that does not appear to have any impact on the morphosyntactic distribution of LIs is not encoded as a semantic feature. For example, that bread is often made with flour is somehow related to the non-linguistic mental representations (concepts) associated with bread but this information is not enshrined in the lexical entry for *bread* as a morphosyntactically potent semantic feature.

There are many ways to encode morphosyntactically relevant semantic features in LIs^12^. For example ^13^, Higginbotham (1989a; see also Glanzberg, 2014, p. 278; Higginbotham, 1989b, p. 167; Ludlow, 2014, p. 99 for discussion) proposes that lexical entries include information concerning what the LI is *true of*; he calls this an *elucidation* of the meaning of a word. For example, the lexical entry for the verb *cut* might include the information in (3), from Higginbotham (1989a, p. 467), a combination of information about thematic structure (the semantic roles patient and agent) and properties related to the action of the verb (i.e., an action that impacts the material integrity of the patient) that appear to have systematic grammatical effects, as illustrated by the middle formation example in (1) above.

(3) “cut” is a V that applies truly to situations *e*, involving a patient *y* and an agent *x* who, by means of some instrument *z*, effects in *e* a linear separation in the material integrity of *y*.

Ludlow (2014, p. 99) observes that some aspects of this lexical entry for *cut* might be stable (such as the thematic structure involving an agent, instrument, and patient), while others (e.g., the notion of linear separation) might be modulated by discourse participants in context. I return to this observation in section 2.3 below.

Summing up the discussion so far, within minimalism LIs are associated with a range of meaning-related properties drawn from multiple areas of cognition. Some but not all meaning-related properties are actually encoded in the lexical entries for LIs as semantic features. An assumption within much of the lexical semantics literature is that semantic features are meaning-related properties that have systematic morphosyntactic effects. An account of the evolution of LIs and the lexicon will need an explicit characterization of what those semantic features are. Without that, we have no foundation for an evolutionary account. Further, accounts of language evolution within minimalism will need to explain the human-unique profile of LIs with respect to meaning. Why do LIs have the semantic features they do? That is the topic of the next section.

### 2.2. The Determination of Semantic Properties

The previous section tried to get a handle on the range of meaning-related properties associated with LIs within minimalism and which of those properties are encoded in the lexicon as semantic features. Different categories of expressions (nominal, verbal, etc.) tend to be associated with different meaning-related properties. Consider the LI *Rihanna*. According to Chomsky (1975, p. 47), from the fact that *Rihanna* is a proper name, it follows that the entity so designated is assigned to “the natural kind Person (hence Animate).” Consequently, the apparent necessity of statements like *The person Rihanna is an animate object* “follows without any attribution of necessary properties to individuals apart from their designation” (Chomsky, 1975, p. 47). Assuming that this claim has some weight to it, we must address the following question: What ultimately determines the semantic features of proper names like *Rihanna*?

This sort of question is what Stalnaker (1997) refers to as a *foundational semantic* question (mentioned in footnote 6 above). Foundational semantic questions are “about what the facts are that give expressions their semantic value” (Stalnaker, 1997, p. 166, 167). In contrast, a *descriptive semantic* question asks what semantic properties expressions have. Kripke (1972) addresses both types of questions with respect to proper names (see Stalnaker, 1997 for discussion). According to Kripke, the semantic value of a proper name like *Rihanna* is its referent (the individual Rihanna), answering the descriptive question *What is the semantic value of “Rihanna”?* This proper name, *Rihanna*, has the semantic value it does because of a particular sort of causal relation between the name and the referent. The identification and description of this causal relation will be part of an answer to the foundational question *Why does “Rihanna” have as its semantic value the individual Rihanna?*

Taylor (2019, p. 43) discusses the transition from descriptive semantic considerations about semantic values and properties to metaphysical considerations about the natures of those values. In a number of publications, Ludlow (1999, 2003, 2011, 2019) has argued that meaningful use of language involves ontological commitments and that there is a strong connection between semantics and metaphysics, proposing that we can use our knowledge of language to “gain insight into the nature of reality” (Ludlow, 1999, p. 179). On Ludlow's view (and many others; see, e.g., Kennedy and Stanley, 2009), semantic theory is about language-world relations; “semantics and metaphysics have to take place hand in hand” (Ludlow, 2019, p. 16). In contrast, Chomsky has argued (see, for example, Chomsky, 1975, 2003b) that study of how expressions of human language relate to extramental individuals, properties, and events will not yield substantive metaphysical theses (except for theses about the language faculty itself), at least in terms of “the enterprise of natural science” (Chomsky, 2003b, p. 289). (Ludlow, 1999, 2003 replies to some of Chomsky's arguments.) Taylor (2019) argues that natural languages are not “fully metaphysically transparent” (p. 30), providing “only shallow initial knowledge into the ultimate metaphysics of the assigned semantic values” (p. 107) and advocates for “metaphysical modesty” in semantics, although he does not claim that language is “completely metaphysically opaque” (p. 30).

Stalnaker (1997, p. 168, 169), in a helpful discussion of the relationship between semantic frameworks and ontology, argues that “the motivation and commitments of [e.g., the possible worlds framework–BC] are more methodological and conceptual than they are metaphysical” (p. 168). Hobbs (1985) argues for ontological promiscuity on the basis that a less plausible (but linguistically faithful) ontology might have theoretical simplicity as a happy byproduct. Similarly, Gross (2015) observes that supposing semantic features of all sorts “might facilitate the modeling and computation of semantic properties and relations.” It doesn't follow from this that the speaker (or the semanticist) is actually committed to the existence in the external world of objects, properties, or events with those properties or involved in those relations. There's much more to be said here, some of which is likely relevant to our understanding of the evolution of words, but I'll set aside questions regarding the relationship between semantics and metaphysics for the remainder of this article.

The goal of semantic theory is typically understood as descriptive (Borg, 2012, p. 160): assign semantic values to LIs and account for how the semantic values of complex expressions are a function of the semantic values of their parts and the way in which those parts are combined (Stalnaker, 1997, p. 166). Semantic theory itself is not (typically understood as being) required to account for the metaphysical character of the semantic properties of LIs. On this conception, semantic theory is required to explain why *Sam smokes* means Sam smokes as a consequence of the semantic value of *Sam* and *smokes*, and the way in which they are combined; it is not expected to tell us why the proper name *Sam* denotes Sam and not Kris or why the verb *smokes* means smokes and not dances.

In contrast, a complete account of language evolution, assuming some form of minimalism, might reasonably be expected to say something about how the meaning-related properties associated with LIs are determined. What makes it the case that certain meaning-related properties of LIs obtain, particularly those properties that are encoded in LIs as morphosyntactically potent semantic features, and not others? Some of the meaning-related properties associated with LIs discussed in the previous section (such as those corresponding to semantic features like CAUSE and AGENT) may have been determined (at least in part) by repeated causal interactions with attributes in the environment during our evolutionary history^14^. Other properties might instead have been fixed primarily by the internal properties of language users or their progenitors, rather than mainly through interactions with features of the extramental environment. Meaning-related lexical properties must be investigated on a case-by-case basis. No single type of account is likely to be explanatory for all semantic features. In the remainder of this section I discuss issues surrounding how to frame foundational semantic questions related to lexical semantics. Let me say up front that while answering these questions may be key to understanding the evolution of words they may not be answerable, directly or indirectly, at least with the evidence available and our current methodological toolkit for addressing language evolution.

Chomsky (see, for example, Chomsky, 1995a, 2000, 2003a,b, 2016) has taken a “strictly internalist, individualist approach to language” (Chomsky, 1995a, p. 13), both foundational and descriptive. Individualist^15^ inquiry of the sort that Chomsky advocates seeks to understand the internal states of an organism, cognitive structures such as the human language faculty (Chomsky, 1995a, p. 27). The individualist approach involves the postulation of mental entities, representations, but individualist inquiry “need not ponder what is represented, seeking some objective construction from sounds or things” (Chomsky, 1995a, p. 53).

Chomsky's position on semantic properties, in particular, is firmly individualistic. Meaning-related lexical properties enter into “interpretation, thought, and action, but there is no reason to seek any other relation to the world” (Chomsky, 1995a, p. 53). The context for Chomsky's individualism is his long-running opposition to what he has called the *referentialist doctrine* (Chomsky, 2016, p. 42). The central tenet of this doctrine, as Chomsky characterizes it, is that there is a direct relation between LIs and extramental entities (e.g., *London* refers to London), as opposed to “things in some kind of mental model, discourse representation, and the like” (Chomsky, 1995a, p. 24). Chomsky has argued that, in contrast to non-human animal communication systems, “natural language has no referential semantics in the sense of relations between symbols and mind-independent entities” (Chomsky, 2016, p. 48). I will not summarize Chomsky's arguments (Chomsky, 2000, 2016, p. 43f.) against the referentialist doctrine, as they have been nicely summarized elsewhere^16^. (Among other things, Chomsky argues that the referentialist doctrine commits us to implausible individuals like *Joe Sixpack* and *John Doe*.) Borg (2012, p. 155; discussing Collins, 2009) observes that it is “the explanatory redundancy of the external dimension to meaning, from the point of view of semantics, which is at the heart of arguments for internalism.”

Looking at the range of properties, such as CAUSE, discussed in the previous section, the meaning-related properties associated with LIs vary in how much of their nature depends constitutively on environmental factors, at least they appear to do so superficially. The determination of at least some of these properties likely involved aspects of the environment of our evolutionarily distant progenitors. Many meaning-related properties externally expressed by linguistic representations appear to be internally represented by some non-human animals. These include the kind BODY, abstract relations like transitivity seemingly grounded in hierarchical social knowledge (e.g., who dominates who), categories and properties of objects (e.g., quality of food and specific predators), discrete numerosities, temporal notions, and spatial notions (see, e.g., Cheney and Seyfarth, 2007; Hurford, 2007; Camp, 2009; Carey, 2009; Burge, 2010).

With the considerations in the previous paragraph in mind, it seems wrong to assume that foundational semantic questions concerning the meaning-related properties associated with LIs, including those enshrined in LIs as morphosyntactically potent semantic features, can and should be given only individualist answers, as Chomsky (1995a, 2000, 2003b, 2016) appears to. As Burge (1989, p. 187; emphasis added—BC) puts it:

Most empirically applicable concepts are fixed by three factors: by actual referents encountered through experience—one's own, one's fellows', or **one's species ancestors'**, or indirectly through theory; by some rudimentary conceptualization of the examples—learned or innately possessed by virtually everyone who comes in contact with the terms; and by perceptual information, inferential capacities, and kind-forming abilities, that may be pre-conceptual.

The individuation of many of the concepts (such as CAUSE, BODY, and ANIMATE) that underpin the semantic properties (both encyclopedic properties and morphosyntactically-relevant ones) that we associate with linguistic expressions likely depend on direct or indirect relations to the extramental environment, by us or our progenitors. An anti-individualist perspective might help us address foundational lexical semantic questions.

The central claim of anti-individualism is that:

The natures of mental states that empirically represent the physical environment depend constitutively on relations between specific aspects of the environment and the individual, including causal relations, which are not in themselves representational; the relevant environment-individual relations help determine specific natures of the states (Burge, 2010, p. 61).

Anti-individualist explanations play a large role in a number of cognitive domains; e.g., visual perception (Burge, 2007a, 2010). The study of visual perception involves the development of empirical theories that are concerned with how visual perception works, seeking to uncover psychological laws. Discussing work on the nature of visual representations and the processes by which they are derived, Chomsky (1995a, p. 52) argues that “the account is completely internalist.” Visual representations, according to Chomsky, are not to be understood relationally, as “representation of” (Chomsky, 1995a, p. 53).

This is an inaccurate characterization of visual perception and its investigation. Burge (2010, p. 98–101; see also Burge, 2003, p. 463–465) agrees that visual psychology as a discipline is primarily focused on explaining processes but argues that the methodology (such as perceptual reports) and the characterization of psychological laws in visual psychology presuppose anti-individualism (i.e., kinds are individuated by representational content)^17^. Environment-individual relations help determine the specific natures of visual representations. The psychological kinds indicated by explanations in visual psychology “can be understood only in an anti-individualistic framework” (Burge, 2010, p. 101). The same is true of the meaning-related representations associated with LIs in lexical semantic work, if our focus is on how those representations are ultimately determined.

Anti-individualism about (certain) semantic properties does not reject the view that meaning is “in” the mind/brain (Burge, 2003, p. 455; see also Burge, 2007b, p. 154; Burge, 2010, p. 64). On an anti-individualist view, the relation between linguistic expressions and semantic values does not make explicit reference to objects, properties, or events in the extramental world. Rather, from an anti-individualist perspective, the natures of certain semantic properties “depend on relations that are not reducible to matters that concern the individual alone. But the natures are not themselves relations, and their representational contents are not themselves (in general) relational” (Burge, 2010, p. 154). While some mental states and their content (semantic properties) are constitutively dependent on relations between the individual and the environment, elements of the environment (entities, properties, or events) are not part of (or part of a relation to) the mental state or the state's representational content. Anti-individualism does not assert a direct connection to the extramental world in the mind/brain of the language user.

Some linguists, such as Jackendoff (2007, p. 353), appear to be confused about this aspect of anti-individualism. Jackendoff has long advocated a “cognitive perspective” on linguistic meaning (see Jackendoff, 2012 for a recent expression of this view), arguing that meanings have to be in the heads of speakers rather than out in the world (Jackendoff, 2012, p. 44). Jackendoff explicitly contrasts his view with the view of anti-individualists like (Putnam, 1975). However, like anti-individualist investigations of word meaning, Jackendoff is interested in explaining how the meaning of a word or sentence, something in the head of a language user, can connect with the world (Jackendoff, 2012, p. 49, 50). Anti-individualism provides us with a framework in which we can develop an answer to this sort of foundational semantic question.

To sum up the discussion so far, some foundational lexical semantic questions (such as how semantic features like CAUSE are determined) likely have anti-individualist answers. (The questions themselves are, in fact, probably coherent only in an anti-individualist framework.) Many meaning-related properties of linguistic expressions appear to be non-individualistically individuated: “What a word means, even in an individual's idiolect, can depend on environmental factors, beyond an individual's body, considered as a molecular structure” (Burge, 1989, p. 178). For example, the nature of semantic features such as CAUSE presumably depend at least partly on the perception of patterns (by us, by our conspecifics, by our evolutionarily distant progenitors) in the environment that are independent of the language faculty^14^.

Some meaning-related properties of LIs are likely the result of causal interactions with the extracranial, distal environment over centuries by one's progenitors (see Burge, 2010, p. 346 for a similar comment regarding how the perceptual system came to mirror environmental regularities). Others may result from linguistic interactions with one's conspecifics during individual development.

The adjustment of lexical meaning during conversation might give us a window into how some meaning-related lexical properties are determined during individual development. Lexical meanings are underdetermined in that “there is no complete answer to what does and doesn't fall within the range of a predicate like ‘red’ or ‘bald”' (Ludlow, 2014, p. 5). The semantic features encoded in lexical entries consist of “just hints and clues … that may help us deploy resources to flesh out word meanings” (Ludlow, 2014, p. 80). There is no privileged core meaning. For example, the meanings of the verb *know* and the noun *knowledge* might be quite a bit more constrained in an epistemology course than in a non-academic conversational context (Ludlow, 2014, p. 5). In some fashion, the lexical entry for the verb *know* encodes that the eventuality it denotes includes an agent and the content of a belief, but contains also “argument places for standards of justification and evidence, for subjective certainty of the report, for the reporter's responsibility for having and defending the knowledge, the source of the knowledge, and the mode of presentation of the content of the knowledge report” (Ludlow, 2014, p. 141, 142). The meaning of *know* is adjustable in context along many different dimensions and ultimately a product of collaborative effort between interlocutors. Ludlow argues that there are norms of word meaning litigation (e.g., “modulations should not be too taxonomically disruptive,”^18^ Ludlow, 2014, p. 48).

We expect the content of anti-individualist explanations to vary depending on the sort of expression we are investigating. The semantic properties of certain types of expressions, e.g., natural kind terms (such as *tiger*) and proper names, may be less closely associated with direct perceptual interactions with the environment, depending more so on the cognitive resources of other members of the social environment than the speaker's perception of external entities, properties, and events (Putnam, 1975; Burge, 1979, 1989, p. 185; Glanzberg, 2018, p. 201).

Anti-individualist work has presented strong arguments that some semantic properties associated with LIs and complex linguistic expressions are constitutively dependent on certain patterns in the social and physical environment “in the evolution of the species as well as in the experiential history of the individual” (Burge, 1989, p. 179). Other semantic properties might instead have primarily individualist explanations. For example, Glanzberg (2018, p. 215) argues that certain verb meanings (e.g., the verb *kill*) might be well-served by an individualist approach, their extensions fixed by theories that speakers represent mentally. Individualism might also give us a better handle than anti-individualism on certain intralinguistic phenomena (e.g., semantic relations like synonymy and polysemy; patterns of syntactic distribution which seem to demand semantic explanation like the middle construction; and verb relations such as that between *persuade* and *intend*). There is no reason to think that lexical properties have an uniformly individualist or uniformly anti-individualist explanation. An anti-individualist explanation may be appropriate for some meaning-related properties of LIs but not others^19^.

To recap, the previous section discussed what semantic properties are associated with LIs, a descriptive semantic question, whereas this section asked how the semantic properties of LIs are determined, a foundational semantic one. An evolutionary account of LIs within minimalism needs to address both questions. But they must be distinguished. It is implausible that all of the semantic features that populate our accounts of word meaning are individuated solely internally without any reference to the external world. Certain, perhaps many, semantic properties are ultimately typed by relations that individuals (us, our conspecifics, our evolutionarily distant progenitors) have borne to their environment. Anti-individualism provides a framework for thinking through what explanations of the constitutive dependence of certain lexical properties on the extramental world might look like, even if the explanatory goals are currently out of reach, given the evidence available, both in practice and perhaps even in principle.

### 2.3. The Structure of Lexical Entries

The previous two sections discussed the taxonomy of lexical semantic features and their grounding. Ultimately, word meanings must “exhibit the format required by the composition operations that correspond to phrasal syntax” (Pietroski, 2010, quoted in Borg, 2012, p. 174). On the assumption that the meaning of a complex expression is determined by the meaning its parts and the way in which those parts of combined—the assumption that natural language meaning is compositional—word meanings must be composable. I'll call this the *compositionality constraint*^20^.

The compositionality constraint will influence our account of the relationship between word meaning and the internal structure of LIs. In the introduction to this section, I characterized the minimalist lexicon as a set of LIs, where each LI is a triple 〈SEM, SYN, PHON〉. SEM and SYN are (possibly empty) subsets of the sets of semantic and syntactic features provided by Universal Grammar, while PHON is a string of segments, possibly null, where each segment is a bundle of features (like VOICE). On this view of LIs, it is non-obvious how to relate an instance of SEM (i.e., a set of features presumably resembling something like, for example, {MANNER OF MOTION, CONTACT, …}) to a semantic value viable within a compositional semantic system like the ones presented in Heim and Kratzer (1998) and Jacobson (2014).

In this section I consider the internal structure of LIs. Borrowing terminology introduced by Glanzberg (2011, 2014, 2018), I discuss how concepts might be *packaged* into lexical entries as semantic features. As discussed earlier, lexical meaning appears to package concepts from a range of cognitive domains as semantic features of LIs. I'll call the process of packaging concepts into LIs as semantic features *lexicalization*^21^. The main goal of this section is to explore what some of our packaging options are and the consequences of these options for our accounts of the evolution of words. I start with a discussion of the conceptual atoms approach advocated for by Fodor in various publications and then turn to a brief case study of Glanzberg's (2011, 2014, 2018) pointers and packaging approach, an approach to lexical semantics that attempts to address the descriptive and foundational semantic questions explored earlier in this article.

Fodor and Lepore (2002, p. 90; see also Fodor, 1998, 2008; Fodor and Pylyshyn, 2015) advocate for conceptual atomism, the view that the semantic component of lexical entries (typically) lacks internal structure, taking this to be a “sort of null hypothesis.” On this view, a lexical entry simply specifies the semantic value (referent) of the corresponding LI rather than specifying, for example, a set of satisfaction conditions, a set of semantic features as in minimalism, or an elucidation of the sort described by Higginbotham (1989a, 1989b) (discussed in section 2.1). For example, according to Fodor and Lepore's view, the semantic component of the lexical entry for *cat* states that *cat* refers to cats (rather than containing, for example, a set of semantic features along the lines of {ANIMAL, …} that gives some indication of lexical meaning); the lexical entry for *Rihanna* states that *Rihanna* refers to Rihanna; the lexical entry for *dance* states that it refers to dancing, etc.

Conceptual atomism fairs quite well with respect to the compositionality constraint, as Fodor (2008, p. 16) argues. Reference is the only mind-world semantic property of the language faculty on this approach (there are no meanings, no senses, etc.). There are just two kinds of reference relations in the system: reference to individuals (by singular terms) and reference to properties (by predicates). As Fodor (2008, p. 199) observes, hardcore “internalists” like Chomsky (see, e.g., Chomsky, 2000) and Jackendoff (see, e.g., Jackendoff, 2012) appear to have an even simpler conception of the semantic component of lexical entries. On their view, lexical entries do not specify semantic values at all (i.e., LIs do not encode mind-world relations), although both Chomsky and Jackendoff assume that LIs are related in some fashion to cognitive structures outside of linguistic competence.

There are several limitations to the conceptual atomist view of the semantic component of lexical entries. First, the conceptual atomist view has no way to account for the claim (discussed in section 2.1) that there are semantic determinants of morphosyntactic distribution (see, e.g., Higginbotham, 1989a; Glanzberg, 2011 for references)^22^. Second, the frugal nature of conceptual atomism does not provide us with any resources to group expressions into different semantic categories (such as a category of manner of motion expressions like *crawl, run, tumble*, …) through semantic properties (Borg, 2012, p. 194)^23^.

To account for the syntactic reflexes of semantic properties and other linguistic phenomena, most approaches to the lexicon (as in minimalism) assume that lexical entries are associated with meaning-related information beyond a simple specification of the LI's semantic value. This is true even of conceptual atomists like Fodor, if you look closely. As discussed in footnote 7 above, Fodor (1998, p. 63) makes a distinction between lexical entries that contain semantic features (i.e., lexical entries that contain bundles of semantic features like SEM) and lexical entries that have meaning-related properties attached to them. Fodor allows for the latter in his atomist view of the lexicon.

Borg (2012, p. 193f.) advocates for a lexicon of the sort that Fodor has in mind, a lexicon comprising lexical entries each of which may have a set of semantic properties attached to them (indicating the semantic class of the LI and any features which affect the LI's syntactic distribution) but possess internally a word-denotation pair (mind-world mapping) alone as their semantic component. For example, the semantic component of the lexical entry for *ready* (as in *Sam is ready*) simply specifies that the referent of *ready* is the property “readiness.” Attached to the lexical entry, though, is additional information about how to construct the logical form of sentences that contain *ready* (Borg, 2012, p. 203). Burge (1989, p. 181) makes a related distinction between a lexical item (what Burge calls “the word”) and “the explication of its meaning that articulates what the individual would give, under some reflection, as his understanding of the word” (what Burge calls the “entry for the word”). Similarly, Burge distinguishes between “the concept associated with the word and the concept(s) associated with the entry”, calling the latter “the conceptual explication” (p. 181).

Glanzberg (2011, 2014, 2018) treats lexical meaning as an interface phenomenon: “semantic competence is only a partial determinant of content” (Glanzberg, 2014, p. 277), at least in the case of lexical vocabulary like nouns and verbs (in contrast to functional vocabulary like quantifying determiners). The semantic component of lexical entries comprises (i) elements of semantic competence and (ii) a pointer to an element in cognition (e.g., a concept) outside of linguistic competence (see Pietroski, 2018 for a somewhat similar view). While lexical entries point to other areas of cognition, they are fully in the faculty of language. Following Glanzberg, I will call this the “pointers and packaging” approach. A key property that distinguishes the pointers and packaging approach from the approaches discussed in the last several paragraphs (e.g., Borg's, 2012 view of the lexicon) is that reference to cognitive structures outside of the language faculty is explicitly encoded within lexical entries through the mechanism of a pointer (rather than, for example, via semantic features attached to lexical entries, as in Fodor's and Borg's characterizations of the lexicon).

Formally, lexical entries split into a structural frame and a pointer. (In what follows, I use italicized capitals to indicate the name of pointers that appear in lexical entries). (4) (from Glanzberg, 2011) gives the semantic component of the lexical entry for the verb *open*. In (4), the structural frame describes the type of event that *open* denotes in terms of a combination of structural elements like CAUSE and BECOME. The pointer “*OPEN*” in (4) indicates the specific, idiosyncratic aspect of the meaning of *open*, pointing to broader conceptual resources outside of linguistic competence^24^. Glanzberg (2011, 2014, 2018) discusses how the pointers and packaging approach fits into a compositional account of semantic competence.

(4) *open*: [[*x* ACT] CAUSE [BECOME *y* 〈*OPEN*〉]]]

The structural frame in lexical entries, like that for *open* in (4), plays an important role in addressing some the issues raised in section 2.1. It gives the grammatically relevant components of lexical meaning, assuming that there are semantic determinants of morphosyntactic distribution (as in middles like *the bread cut easily* and resultatives like *Sam pounded the metal flat*). With other work in lexical semantics (see Levin and Hovav, 2005), the pointers and packaging approach assumes that there is a finite set of structural elements like CAUSE and BECOME and that there are constraints on how these structural elements can be combined.

Glanzberg (2011) discusses the nature of structural elements like CAUSE that appear in lexical semantic representations. These elements are part of the language faculty proper. Consequently, the element CAUSE, for example, is not to be identified with the word *cause* or the intuitive concept of causation. There is solid evidence against identifications of this sort. For example, it has long been observed that CAUSE (argued to be a component of the lexical semantic representations of the meanings of verbs like *break* and *open*) is more restricted than the intuitive concept of causation (e.g., Dowty, 1979). Compare (modifying a minimal pair presented in Glanzberg, 2011): *I caused the glass to break, by paying Sam to throw it against the wall* and #*I broke the glass, by paying Sam to throw it against the wall*, suggesting that CAUSE (when a component of the structural frame for a verb like *break*) expresses something akin to direct causation.

Pointers give LIs their distinctive content, pointing to mental representations that live outside of the faculty of language. They are the source of the encyclopedic, worldly information associated with LIs. The pointers and packaging model, as such, is not susceptible to some of the same criticisms that Fodor (1998, 2008; also Fodor and Lepore, 2002) presents against decompositional/definitional approaches to concepts. The extralinguistic concepts that LIs interface with through pointers are linguistically atomic, at least as far as the theoretical characterization of semantic competence is concerned (Glanzberg, 2014, p. 282, 284)^25^.

The pointers and packaging approach also provides us with a way to capture Rips's (2011, p. 163–164) distinction between representation about and representation of a category. Mental representation about a category (like towel, padlock, or daisy) gives all the information we have about the category, whereas mental representation of a category is just an unchanging atomic symbol. The pointer “*OPEN*” in the structural frame for *open* in (4) is a mental representation of whatever (complex or simple) outside of language cognition corresponds to the idiosyncratic aspect of the meaning of *open*.

To review, within minimalism, lexical entries are internally complex, containing semantic, phonological, and syntactic information. Lexical meaning itself is multidimensional. On the one hand, LIs typically express idiosyncratic content distinct from that of other LIs. On the other hand, LIs appear to be associated with semantic features that, among other things, influence their morphosyntactic distribution. The pointers and packaging approach is one way to organize these dimensions within lexical entries and address the compositionality constraint discussed at the beginning of this section.

From the standpoint of a minimalist account of the evolution of words, though, a lexicon consisting of internally structured lexical entries presents a challenging puzzle, whether the structure of those entries is a triple 〈SEM, SYN, PHON〉 of the sort assumed by much work in minimalism or has the form proposed in the pointers and packaging approach. As discussed in the introduction to this section, many non-human animals appear to have concepts and some of these concepts appear to be similar to those that populate human cognition. But the signals that populate animal communication systems (like predator-specific alarm calls) do not appear to have anything like the internal structure of LIs nor do they express similar content. Accounting for the emergence of internally complex LIs is a significant open problem in our understanding of language evolution.

## 3. Where Now?

In the introduction I mentioned the *criterion of evolvability*: “any mechanisms and primitives ascribed to UG rather than derived from independent factors must plausibly have emerged in what appears to have been a unique and relatively sudden event on the evolutionary timescale” (Chomsky et al., 2017). This criterion imposes limitations on our account of LIs and the lexicon. Minimalist approaches to LIs (of the sort reviewed here) assume that LIs have a complex internal structure, consisting of three set of features (phonological, semantic, and syntactic). If we focus our attention on the semantic properties associated with LIs, it's quite possible that lexical entries are even more complex than the view that I presented in the introduction indicates. It's not clear how to reconcile this with the criterion of evolvability^26^.

In the main body of this article I addressed three questions: what (meaning-related) properties are associated with LIs, assuming a minimalist view of the human language faculty, how are those properties determined, and what is the internal structure of lexical entries? A range of properties appear to be associated with LIs, but not all of those properties are encoded in the lexicon as semantic features. Work on lexical semantics suggests that semantic features should be limited to features that affect the morphosyntactic distribution of the corresponding LIs. Distinguishing between descriptive semantic and foundational semantic questions, and anti-individualist and individualist answers, provides a way of thinking about what questions we might ask about the nature of those features (e.g., CAUSE) within the context of language evolution. The pointers and packaging approach to the lexicon suggests how we might couple semantic features with a mechanism that accounts for the distinctive content of individual LIs and the observation that lexical meaning is an interface phenomenon, while maintaining a relatively simple conception of the lexicon. Giving some thought to how this approach to the lexicon fits into a broader account of language evolution might move us a step closer to understanding what we can and cannot learn about the evolution of our capacity for language.

## Author's Note

I have profited from the comments of Noam Chomsky and three reviewers for *Frontiers in Psychology*. Errors and obfuscations remain my own.

## Author Contributions

The author confirms being the sole contributor of this work and has approved it for publication.

### Conflict of Interest

The author declares that the research was conducted in the absence of any commercial or financial relationships that could be construed as a potential conflict of interest.

## Footnotes

^1^My use of the term *minimalism* throughout this article should be distinguished from the use of *minimalism* to refer to minimal semantics (Borg, 2004, 2012), a particular approach to semantic theorizing. Minimal semantics is, very roughly, committed to the view that well-formed (declarative) sentences express truth-evaluable content and that content is fully determined by syntactic structure and lexical content. Although minimal semantics assumes that there are (a limited number of) context-sensitive expressions, the input of context to literal content is taken to be severely constrained (Borg, 2012, p. 4, 5). The tenets of minimal semantics intersect in an interesting way with the goals of the minimalist program. I will not explore this intersection here, but I return to Borg's work in my discussion below of the structure of lexical entries.

^2^The order of presentation here of these developments is not intended to indicate a claim about their relative ordering during human evolution.

^3^Marler et al. (1992) present two criteria that must be met for a signal to be functionally referential: • Production criterion: all the stimuli that elicit the signal belong to one category, either a general category such as “aerial predators” or a more specific one such as “eagle”. • Perception criterion: the utterance of the referential signal is alone sufficient to elicit the same behavior as would be elicited by perceiving the referent.

^4^While features play a central, indispensable role in minimalism and a great deal of other work in linguistics, there is little agreement on what features there are and their key properties. For this reason, I make no attempt in what follows to present a complete inventory of lexical semantic features. My goal here, among others, is simply to try to get a handle on what kinds of lexical semantic features have been associated with LIs. Adger (2010) and Adger and Svenonius (2011) discuss many of the major issues concerning features and feature structures in minimalism.

^5^Focusing on semantic properties of LIs, I take this (the question of how the properties of LIs are determined) to be a question of foundational semantics, drawing on Stalnaker's (1997) distinction between descriptive semantic questions (e.g., what is the semantic value of the LI *Rihanna*?) and foundational semantic questions (e.g., what makes it the case that the lexical item *Rihanna* has the semantic properties that it has?). I return to this distinction below.

^6^Prinz (2002) argues that all human concepts have a perceptual basis. Rips (2011) presents a range of evidence that concepts include information that goes beyond what purely perceptual mechanisms afford and that non-perceptual modes of thought are central to basic cognitive notions such as numbers and causality. As I discuss below, Fodor argues in a number of publications (see, e.g., Fodor, 1998; Fodor and Lepore, 2002) for conceptual atomism, the view that most concepts have no internal structure (discussed below). Prinz (2002) argues against atomism, primarily on the grounds that atomic representations can't explain our capacity to categorize because these representations do not contain features. Fodor (1998, p. 63) observes that the claim that an element (a concept, an LI) has features is distinct from the claim that the element is a feature bundle or comprises multiple sets of features (as in minimalism). This distinction reduces the force of Prinz's argument.

^7^Many thanks to a reviewer for pointing me to Tallerman's article.

^8^Harley (2010) presents an overview of different minimalist treatments of argument structure phenomena (i.e., linguistic phenomena that involve the morphosyntactic realization of the core participants in the eventuality an LI like *break* denotes). Some of these treatments dispense with argument structure (as a feature of LIs) entirely, while other treatments have preserved the traditional assumption that semantic roles (like AGENT) are associated with lexical features of some sort.

^9^For example, Chomsky (1975, p. 233) stresses that there might be analytic connections (for example, between *persuade* and *intend*: “*x* persuade *y* to *z*” entails “*y* intend to *z*”) that can be accounted for “by virtue of the substructure of lexical features and their general properties”, features such as CAUSE, BECOMING, AGENCY, and GOAL.

^10^Glanzberg (2011) distinguishes two sorts of concepts that run in parallel, the linguistic meaning of an LI (encoded representationally in the lexical entry for the LI) and the non-linguistic mental representations that are associated with the LI. He speculates “that a substantial amount of our most sophisticated thinking makes direct use of the meanings of lexical items, rather than the associated non-linguistic concepts.” As noted above, certain apparent differences between the conceptual repertoires of human and non-human animals may be a byproduct of lexicalizing concepts as components of LIs, rather than differences in non-linguistic conceptual resources across species (“public use affects private concepts”).

^11^Fodor and Lepore (2002, p. 99–102) dispute the claim that there are semantic determinants of morphosyntactic distribution; see Fodor (1998, p. 56–64) for related discussion.

^12^In a number of publications, Fodor (e.g., Fodor, 1998, p. 59, Fodor, 2008, p. 28; Fodor and Lepore, 2002, p. 99; see Borg, 2012, p. 187 for an overview of Fodor's critique) criticizes the use of theoretical vocabulary like CAUSE by lexical semanticists because, among other things, the semantics of this vocabulary is typically unspecified: “It is … notoriously difficult to assess the claimed correlations between lexical semantics and syntactic distribution, because one is never told what the semantic representations themselves mean” (Fodor and Lepore, 2002, p. 99). According to this argument, without a specification of what CAUSE, AGENT, ACT, etc. denote it is not possible to assess whether a particular proposal involving this sort of vocabulary explains the morphosyntactic phenomenon in question (e.g., the middle construction). Although the features that populate semantic representations in lexical semantics work are frequently left undefined, the claim that “one is never told what the semantic representations themselves mean” [emphasis added—BC] is false, though, as Glanzberg (2014, p. 278) points out. For two exceptions (among others) to Fodor and Lepore's claim, see Dowty (1979), who presents an explicit semantics for CAUSE and BECOME (and much else), and Rothstein (2004), who also presents an analysis of BECOME (and much else).

^13^Levin and Hovav (2005) is a comprehensive survey of different approaches to lexical semantic representation. Glanzberg (2018, p. 207, 208) provides further references to a variety of approaches to lexical semantics.

^14^But Rips (2011) presents evidence that perceptual information alone is not enough to ground notions like causality. (See footnote 6 above.)

^15^*Individualist* (*individualism, individual*) is used seemingly interchangeably with *internalist* (*internalism, internal*), and *anti-individualist* (*anti-individualism, anti-individual*) with *externalist* (*externalism*, external) in the philosophical literature that I am familiar with. An explicit distinction between (analogs to) individualism and anti-individualism seems to be rarely made in the linguistics literature, mostly likely because descriptive concerns are often primary. I use the terms *individualist* (*individualism, individual*) and *anti-individualist* (*anti-individualism, anti-individual*) in what follows for reasons discussed by Burge (2007b, p. 154).

^16^See especially Ludlow (1999, Appendix P2, 2003) and Stainton (2006).

^17^Kennedy and Stanley (2009) make a similar remark about the methodology of natural language semantics, although they do not directly discuss externalism/anti-individualism.

^18^Ludlow (2014, p. 41–51) proposes this particular norm of word meaning litigation in the context of a discussion of the word *planet*. See also Jackendoff (2012, p. 60, 61) on *planet*.

^19^Fodor (1998) speculates about how interactions between individuals and the environment might result in the acquisition of concepts that can be labeled, proposing what he calls the *locking model of concept possession* (see also Fodor, 2008). Stainton and Viger (2000) present a helpful exegesis of Fodor's model (see also Borg, 2012, p. 195 for brief discussion). Roughly, an individual acquires a concept when a form (a neural structure) within a person's brain becomes “locked” to an extramental entity, property, or event “through brute causal interaction with the environment” (Stainton and Viger, 2000, p. 142). On this view, there are few, if any, innate concepts (cf. Fodor, 1975). Prinz (2002, p. 228–235) critiques Fodor's accounts of concept acquisition. Carey (2009) and Rips (2011) both contain thoroughgoing discussions of concept acquisition.

^20^I think that I'm borrowing the name of this constraint from Davidson by way of Fodor and Lepore.

^21^The term *lexicalization* has a range of more-or-less related uses in the language evolution and historical linguistics literature. It has sometimes been used to refer to the synchronic process of associating concepts/conceptual structure with forms (sounds, gestures, etc.) to create symbols. Boeckx (2011, p. 53) describes lexicalization as a key step in the evolution of syntax: the endowment of concepts with properties (so-called *edge features*) that make them active syntactically, combinable with virtually any other concept. Within the historical linguistics literature (see, for example, Brinton and Traugott, 2005), *lexicalization* has been broadly defined as a diachronic process involving the addition of LIs to the lexicon.

^22^Although, as I pointed out earlier, Fodor (1998) and Fodor and Lepore (2002, p. 99–102) dispute the claim that there are semantic properties of this sort. Hence, on their view, there is no need for lexical semantic features (e.g., CAUSE) like those proposed by lexical semanticists. The challenge for this position is to then account for the massive lexical semantics literature that suggests otherwise.

^23^Fodor and Lepore (2002) ultimately settle, however, on a more complex structure for the semantic component of lexical entries. In addition to specifying the referent of the LI, some lexical entries include a composition rule that plays a role in determining the logical form of phrases of which the LI is a constituent (Fodor and Lepore, 2002, p. 113). For example, the lexical entry for *want* contains a composition rule that ensures that the compositional semantics assigns the interpretation ‘wants to have NP’ (e.g., “wants to have a drink”) to phrases of the form *wants NP* (e.g., *wants a drink*).

^24^Glanzberg (2014, p. 281) observes that “there is nothing particularly internalist about this interface picture.” Whatever pointers point to elsewhere in cognition must ultimately play a role in providing a semantic value for the corresponding LI. An explanatory account of how this aspect of the content of an LI is determined could be individualist or anti-individualist depending on the target of the pointer.

^25^Wellwood (2019, p. 194) observes a potential limitation of the pointers and packaging approach. If pointers connect LIs to domain-specific concepts outside of the language faculty, then how does the generality that human language affords thought emerge? As Wellwood puts it (p. 194): “If all we supposed was that linguistic meanings link pieces of syntax with concepts that, in many cases, are domain-specific and isolated from other ones, it would be difficult to see how that kind of generality could ever emerge.”

^26^On the assumption that an account of the evolution of words must satisfy this criterion.
